# Iron dictates the growth, biofilm formation, and virulence of *Pseudomonas aeruginosa* in pulmonary infections

**DOI:** 10.3389/fmicb.2025.1742683

**Published:** 2026-01-16

**Authors:** Chao An, Ruan Chen, Baijian Wu, Shanjian Chen, Shenghua Zou, Yulan Lin, Bin Yang, Chenshuo Luo

**Affiliations:** 1 Department of Laboratory Medicine, The First Affiliated Hospital, Fujian Medical University, Fuzhou, China; 2 Department of Laboratory Medicine, National Regional Medical Center, Binhai Campus of the First Affiliated Hospital, Fujian Medical University, Fuzhou, China; 3 Fujian Key Laboratory of Laboratory Medicine, The First Affiliated Hospital, Fujian Medical University, Fuzhou, China; 4 Clinical Laboratory Diagnostics, The First Clinical College, Fujian Medical University, Fuzhou, China; 5 Department of Orthopaedic Surgery, The First Affiliated Hospital, Fujian Medical University, Fuzhou, China; 6 Department of Laboratory Medicine, Fuzhou Pulmonary Hospital of Fujian Province, Fuzhou, China

**Keywords:** biofilm, growth, iron, *Pseudomonas aeruginosa*, virulence

## Abstract

**Background:**

*Pseudomonas aeruginosa* is the most prevalent pathogen responsible for persistent pulmonary infections. Iron concentrations in the human lung are known to vary considerably between health and disease states. We hypothesized that increased iron availability is a key driver of persistent infection establishment and sought to define the impact of iron on *P. aeruginosa* in the context of persistent lung infection.

**Methods:**

Clinically isolated strains of *P. aeruginosa* from persistent lung infections and the reference strain PAO1 were collected. We examined bacterial growth rates, virulence determinants such as biofilm formation and pyocyanin production, and adhesion to lung epithelial cells under different iron conditions. Virulence was assessed using a *Galleria mellonella* model, and pathogenicity was evaluated in a mouse model.

**Results:**

Compared to *P. aeruginosa* grown in iron-deficient medium, bacteria cultured in iron-rich medium exhibited significantly enhanced growth rates and biofilm formation, while virulence determinants were attenuated. The *Galleria mellonella* model also showed reduced virulence. Additionally, iron-rich conditions enhanced bacterial adhesion to lung epithelial cells. In the mouse model, weakened pathological damage and higher bacterial loads in the lungs were observed.

**Conclusion:**

Our findings indicate that environmental iron facilitates the growth and biofilm formation of *P. aeruginosa* causing pulmonary infections, while attenuating its virulence. This iron-mediated adaptation may be associated with the persistence of *P. aeruginosa* pulmonary infections, and these findings merit further investigation.

## Introduction

*Pseudomonas aeruginosa* (*P. aeruginosa*) is a common opportunistic pathogen in clinical settings, capable of causing wound infections, respiratory tract infections, urinary tract infections, and other conditions (Pont et al., 2025; Faure et al., 2018; Sorbets et al., 2019). Among these, persistent pulmonary infections caused by *P. aeruginosa* represent one of the most challenging clinical conditions to manage. A biofilm is a structured community of microbial cells enclosed in a self-produced polymeric matrix that adheres to a surface (Preda and Săndulescu, 2019). *P. aeruginosa* serves as a model organism for studying bacterial biofilms. The formation of biofilms enhances the ability of *P. aeruginosa* to adapt to external environments and is a major factor contributing to the recalcitrance of persistent lung infections to treatment (Jones and Wozniak, 2017).

The virulence of *P. aeruginosa* is mediated by an array of determinants, including siderophores, proteases, alkaline protease, flagella, pyocyanin, elastase, hemolysins, phospholipase C, exotoxin A, rhamnolipids, among others (Liao et al., 2022). These virulence factors have been demonstrated to play pivotal roles in the pathogenesis of infectious diseases caused by *P. aeruginosa* (Schaible et al., 2017). For example, exotoxin A targets eukaryotic elongation factor 2 (eEF-2), inhibiting host protein synthesis and inducing host cell death (Du et al., 2010). Pyocyanin causes infection by inducing oxidative stress in host cells and disrupting the host’s immune function (Lau et al., 2004). Extracellular invasive enzymes, including elastase, alkaline protease, and protease, degrade host connective tissue, leading to lung parenchymal damage and hemorrhage, and are associated with disseminated infections by *P. aeruginosa* (Sánchez-Jiménez et al., 2023).

*P. aeruginosa* possesses the ability to continuously sense and respond to environmental cues such as bicarbonate levels, osmotic stress, pH, and iron availability (Ruksakiet et al., 2021). Among these factors, iron stands out as one of the most critical elements. Previous studies have reported that iron plays an essential role in both biofilm formation and virulence expression of *P. aeruginosa* (Hamza et al., 2023; Banin et al., 2005; Minandri et al., 2016). However, host-bound iron remains largely inaccessible to most microorganisms, including *P. aeruginosa.* To overcome this limitation, the pathogen employs siderophore production and secretion to acquire iron necessary for growth and metabolism (Mayneris-Perxachs et al., 2022). These iron-chelating molecules not only facilitate iron acquisition and support infection establishment but also significantly influence bacterial survival within the host.

It has been demonstrated that the urogenital tract environment significantly influences the growth and virulence factor levels of *P. aeruginosa* and may contribute to difficulties in treating chronic infections (Subramaniyan et al., 2025). While bioavailable iron in the human body is strictly limited, the pulmonary environment exposes *P. aeruginosa* to varying levels of iron, an environmental factor that may impact its virulence and pathogenicity (Haas et al., 2023). Therefore, it is necessary to understand the precise role of iron in *P. aeruginosa*-mediated persistent pulmonary infections. Current research combining *in vitro* and *in vivo* experiments to investigate the role of iron in *P. aeruginosa* persistent pulmonary infection remains scarce. Our study aims to assess, using both *in vivo* and *in vitro* models, whether varying iron concentrations affect the growth, biofilm formation, and virulence of *P. aeruginosa* that causes persistent pulmonary infection.

## Materials and methods

### Bacterial strains

Two clinical isolates of *P. aeruginosa* (designated PA1 and PA3) obtained from sputum samples of patients with persistent pulmonary infections at The First Affiliated Hospital of Fujian Medical University in 2025, along with the reference strain PAO1 purchased from China Bosa Biological Co., Ltd., were selected for this study. These strains are capable of producing most recognized virulence determinants, including siderophores, proteases, hemolysins, pyocyanin, among others.

### Effect of iron

We referenced previous studies on the influence of iron on the pathogenicity of *Klebsiella pneumoniae* (Chen et al., 2020). As shown in the Supplementary Figure 1, we first confirmed the growth curve of the *P. aeruginosa* reference strain PAO1 under different iron conditions using a microplate reader. We created varying iron-deficient conditions by supplementing TSB with 0 μM, 100 μM, 200 μM, 300 μM, 400 μM, and 500 μM of the iron chelator dipyridyl (DP). With the increasing concentration of DP, the growth of PAO1 gradually slowed down. At 500 μM DP, PAO1 exhibited almost no growth. Therefore, we selected TSB supplemented with 400 μM DP as the most stringent iron-deficient condition. To confirm that the growth inhibition of *P. aeruginosa* PAO1 in TSB supplemented with 400 μM DP, representing the most stringent iron-deficient condition, was specifically due to iron limitation, we added FeCl₃ at concentrations of 0 μM, 0.1 μM, 1 μM, 5 μM, and 10 μM. As shown in Supplementary Figure 2, the growth of PAO1 gradually recovered with increasing FeCl₃ supplementation. When 10 μM FeCl₃ was added, the growth of PAO1 did not show a significant further increase compared to 5 μM. Therefore, for further experiments, we selected the condition of TSB supplemented with 400 μM DP (the most stringent iron-deficient medium) with an additional 5 μM FeCl₃. We measured the iron concentration and growth curves of the collected *P. aeruginosa* in TSB medium supplemented with 0 μM DP, 400 μM DP, and 400 μM DP + 5 μM FeCl₃.

### Determination of iron concentrations

The iron concentration in TSB media containing 0 μM DP, 400 μM DP, and 400 μM DP + 5 μM FeCl₃ was determined using a colorimetric chelation assay. Briefly, ascorbic acid was added to the medium and reacted with Ferene to form a blue complex, which was quantified spectrophotometrically.

### Growth curves

The assessment of iron’s influence on *P. aeruginosa* growth was conducted with modifications based on a previously established methodology (Chen et al., 2020). Briefly, PA1, PA3, and PAO1 strains were cultured overnight in TSB and harvested. The overnight cultures were diluted 1:100 in TSB media containing different iron concentrations: 0 μM DP, 400 μM DP, and 400 μM DP supplemented with 5 μM FeCl₃. Cultures were incubated at 37 °C with constant shaking at 220 rpm. Optical density at 600 nm (OD_600_) was measured at 2-h intervals over a 24-h period. Analysis was based on the average absorbance value obtained from triplicate measurements for each sample.

### Biofilm formation assay

The effect of iron on *P. aeruginosa* biofilm formation was evaluated using a modified established methodology (Chen et al., 2020). Overnight cultures of strains PA1, PA3, and PAO1 in TSB medium were diluted 1:100 in TSB containing different iron conditions (0 μM DP, 400 μM DP, and 400 μM DP + 5 μM FeCl₃). A 200 μL aliquot of each dilution was added to a 96-well polystyrene microtiter plate and incubated at 37 °C for 24 h, with sterile TSB serving as a negative control. Following incubation, the wells were washed thrice with PBS (pH 7.0), air-dried, fixed with methanol for 20 min, stained with 1% crystal violet for 15 min, and then rinsed with PBS until colorless. After drying, the bound dye was solubilized with 200 μL absolute ethanol, transferred to a new plate, and the absorbance was measured at 570 nm. Analysis was based on the average absorbance value obtained from triplicate measurements for each sample.

### Cell-free supernatant and bacterial pellet

Overnight cultures of *P. aeruginosa* strains PA1, PA3, and PAO1 grown in TSB medium were diluted 1:100 in TSB media containing varying iron concentrations (0 μM DP, 400 μM DP, and 400 μM DP + 5 μM FeCl₃). The diluted bacterial suspensions were incubated at 37 °C with constant shaking at 180 rpm for 48 h. After adjusting the cultures to an OD_600_ of 1.0, the samples were centrifuged at 5,000 rpm for 5 min to collect both cell-free supernatants and bacterial pellets.

### Pyocyanin production

A 5 mL aliquot of the collected *P. aeruginosa* cell-free supernatant was mixed with 3 mL of chloroform. The resulting mixture was vortexed and centrifuged. The upper aqueous layer was then treated with 1 mL of 0.2 M hydrochloric acid for acidification. Following thorough mixing and subsequent centrifugation, the upper pink phase was carefully collected, and its absorbance was measured at 520 nm. Analysis was based on the average absorbance value obtained from triplicate measurements for each sample.

### Hemolysin production

Hemolytic activity was measured using a modified version of the method described by previous Research (Alamu et al., 2022; Wang et al., 2022). Briefly, a 5% suspension of sheep red blood cells (obtained from Nanjing Senbeijia Biological Technology Co., Ltd.) was incubated with an equal volume of cell-free supernatant harvested from *P. aeruginosa* culture. The mixture was incubated at 37 °C for 1 h, followed by centrifugation at 500 × g for 5 min. The absorbance of the resulting supernatant was measured at 450 nm. Analysis was based on the average absorbance value obtained from triplicate measurements for each sample.

### Siderophore production

Following the manufacturer’s instructions, the Chrome Azurol S (CAS) assay solution (Beijing Coolaber Technology Co., Ltd.) was mixed with an equal volume of either the collected *P. aeruginosa* cell-free supernatant or deionized water. The mixtures were protected from light and incubated at room temperature for 1 h. The absorbance of the supernatant mixture (A) and the deionized water mixture (Ar) was then measured at 630 nm. Siderophores induce a color change in the CAS assay solution, resulting in a decrease in OD_630_. Therefore, siderophore production was quantified using the following formula: 1 − A/Ar. Analysis was based on the average absorbance value obtained from triplicate measurements for each sample.

### Elastase production

Briefly, *P. aeruginosa* cells cultured under different iron conditions were collected by centrifugation (5,000 rpm, 5 min). The supernatant was discarded, and any residual liquid was thoroughly removed. The corresponding extraction buffer was added at a ratio of 10 μL per 10^7 cells, followed by gentle mixing via pipetting. The cells were then lysed using ice-bath sonication (power 300 W, 3 s pulse on, 7 s pulse off, total duration 3 min). The lysate was centrifuged at 8000 g for 10 min at 4 °C, and the resulting supernatant was collected and kept on ice for subsequent detection. According to the manufacturer’s instructions, the elastase activity in the supernatant was measured using the EnzChek Elastase Assay Kit. Analysis was based on the average absorbance value obtained from triplicate measurements for each sample.

### Protease production

Briefly, *P. aeruginosa* cells cultured under different iron conditions were collected by centrifugation (5,000 rpm, 5 min). The supernatant was discarded, and any residual liquid was thoroughly removed. The corresponding extraction buffer was added at a ratio of 10 μL per 10^7 cells, followed by gentle mixing via pipetting. The cells were then lysed using ice-bath sonication (power 300 W, 3 s pulse on, 7 s pulse off, total duration 3 min). The lysate was centrifuged at 8000 g for 10 min at 4 °C, and the resulting supernatant was collected and kept on ice for subsequent detection. According to the manufacturer’s instructions, the protease activity in the supernatant was measured using the Protease Assay Kit from Beyotime Biotechnology Co., Ltd. Analysis was based on the average absorbance value obtained from triplicate measurements for each sample.

### Alkaline protease production

Briefly, *P. aeruginosa* cells cultured under different iron conditions were collected by centrifugation (5,000 rpm, 5 min). The supernatant was discarded, and any residual liquid was thoroughly removed. The corresponding extraction buffer was added at a ratio of 10 μL per 10^7 cells, followed by gentle mixing via pipetting. The cells were then lysed using ice-bath sonication (power 300 W, 3 s pulse on, 7 s pulse off, total duration 3 min). The lysate was centrifuged at 8000 g for 10 min at 4 °C, and the resulting supernatant was collected and kept on ice for subsequent detection. According to the manufacturer’s instructions, the alkaline protease activity in the supernatant was measured using the Alkaline Protease Assay Kit from Box Biotechnology Co., Ltd. The results were normalized to the total protein concentration in each sample using an Enhanced BCA Protein Assay Kit (Beyotime). Analysis was based on the average absorbance value obtained from triplicate measurements for each sample.

### Phospholipase C production

Briefly, *P. aeruginosa* cells cultured under different iron conditions were collected by centrifugation (5,000 rpm, 5 min). The supernatant was discarded, and any residual liquid was thoroughly removed. The corresponding extraction buffer was added at a ratio of 10 μL per 10^7 cells, followed by gentle mixing via pipetting. The cells were then lysed using ice-bath sonication (power 300 W, 3 s pulse on, 7 s pulse off, total duration 3 min). The lysate was centrifuged at 8000 g for 10 min at 4 °C, and the resulting supernatant was collected and kept on ice for subsequent detection. According to the manufacturer’s instructions, the phospholipase C activity in the supernatant was measured using the Phospholipase C Assay Kit from Elabscience Biotechnology Co., Ltd., and the total protein concentration in each sample was normalized using the Enhanced BCA Protein Assay Kit (Beyotime). Analysis was based on the average absorbance value obtained from triplicate measurements for each sample.

### Exotoxin A production

Briefly, *P. aeruginosa* cells cultured under different iron conditions were collected by centrifugation (5,000 rpm, 5 min). The supernatant was discarded, and any residual liquid was thoroughly removed. The corresponding extraction buffer was added at a ratio of 10 μL per 10^7 cells, followed by gentle mixing via pipetting. The cells were then lysed using ice-bath sonication (power 300 W, 3 s pulse on, 7 s pulse off, total duration 3 min). The lysate was centrifuged at 8000 g for 10 min at 4 °C, and the resulting supernatant was collected and kept on ice for subsequent detection. According to the manufacturer’s instructions, the production of exotoxin A in the supernatant was measured using the Exotoxin A Assay Kit from Wuhan Fine Biotech Co., Ltd. Analysis was based on the average absorbance value obtained from triplicate measurements for each sample.

### *Galleria mellonella* infection model

The larval lethality assay using *Galleria mellonella* was performed with modifications based on a previously established method (Chen et al., 2020). Larvae were anesthetized on ice for 5 min. Bacterial pellets of *P. aeruginosa*, prepared as described above, were resuspended in phosphate-buffered saline to achieve a concentration of 10^8^ CFU/mL. A 25 μL Hamilton microsyringe was used to inject 10 μL of the bacterial suspension (10^8^ CFU/mL) into the last left proleg of each larva. Inoculated larvae were incubated in darkness at 25 °C and monitored at 24, 48, and 72 h post-infection. Larvae were considered dead when they displayed no response to repeated physical stimulation. Results were analyzed using the Kaplan–Meier method.

### Lung epithelial cell adhesion assay

The Lung epithelial cell adhesion assay was conducted with modifications based on a previously described method (Liu et al., 2023). Human Normal Lung Epithelial Cells (BEAS-2B cells) were purchased from Shanghai Biyuntian Biotechnology Co., Ltd. BEAS-2B cells were cultured in high-glucose Dulbecco’s Modified Eagle Medium (DMEM) supplemented with 10% fetal bovine serum at 37 °C under 5% CO₂. The prepared *P. aeruginosa* bacterial pellets were resuspended in DMEM and added to 24-well plates at a multiplicity of infection (MOI) of 10. After 2 h of incubation, the supernatant was discarded, and the cells were washed with sterile PBS to remove non-adherent bacteria. The cells were then lysed with 0.01% Triton X-100, and the lysates were plated on blood agar plates for bacterial enumeration. Analysis was based on the average absorbance value obtained from triplicate measurements for each sample.

### Mice infection model

The mice lung infection model was established with modifications based on a previously described methodology (Nielsen et al., 2018). Male C57BL/6 mice (6–8 weeks old) were obtained from Beijing Huafukang Biological Technology Co., Ltd. The mice were anesthetized using isoflurane (Shanghai Yuyan Instruments Co. Ltd., Shanghai, China), and their upper incisors were secured with fine thread to suspend them in a vertical position on a rack. The tongue was carefully extended from the oral cavity using sterile blunt forceps and gently immobilized. The nasal passages were temporarily occluded to maintain oral breathing. Concurrently, a second operator administered 50 μL of a bacterial suspension containing *P. aeruginosa* pellets resuspended in PBS (1 × 10^9^ CFU/mL) at the oropharyngeal junction using a micropipette. Control mice received an equal volume of PBS buffer. Successful pulmonary inoculation was confirmed by a characteristic crackling sound upon reflex inhalation, indicating aerosolization and lung deposition of the inoculum. At 24 h post-infection, mice were anesthetized with isoflurane and sacrificed by cervical dislocation for subsequent lung tissue isolation.

### Lung tissue isolation

Mice lung tissues were aseptically harvested and rinsed with PBS, followed by weighing with an analytical balance to record tissue mass. The left lung was placed in a microcentrifuge tube containing 500 μL of pre-cooled saline. The tube was maintained on ice, and tissue homogenization was performed using a mechanical homogenizer with five cycles of 60-s grinding interspersed with 10-s intervals. The resulting homogenate was utilized for bacterial enumeration and ELISA-based quantification of inflammatory cytokine levels. The right lung was transferred to a 2 mL microcentrifuge tube and fixed with 4% paraformaldehyde at room temperature for 48 h in preparation for pathological examination.

### Lung bacterial load

A 100 μL aliquot of the lung tissue homogenate was serially diluted in sterile PBS, and 50 μL of the appropriate dilution was plated onto blood agar plates. After spreading with a sterile cell spreader and air-drying, the plates were incubated at 37 °C overnight. Bacterial colonies were enumerated, and the bacterial load in lung tissue was calculated as CFU per gram of tissue. All experiments were performed in triplicate.

### Lung tissue cytokine analysis

The levels of interleukin-6 (IL-6), interleukin-10 (IL-10), and procalcitonin (PCT) in the lung homogenates were quantified using commercial ELISA kits (Elabscience Biotechnology Co., Ltd.) according to the manufacturer’s protocols. Analysis was based on the average absorbance value obtained from triplicate measurements for each sample.

### Lung histopathological examination

The fixed lung tissues were embedded in paraffin after overnight fixation in 4% paraformaldehyde. Sections of 5 μm thickness were prepared and stained with hematoxylin and eosin (H&E) for assessment of pulmonary damage severity (Sen-Kilic et al., 2019).

### Statistical analysis

Data were analyzed using analysis of variance (ANOVA), Fisher’s exact test, and Student’s t-test. A *p*-value of less than 0.05 was considered statistically significant. All statistical analyses were performed with SPSS version 19.0.

## Results

### Determination of iron concentrations in culture media

To investigate the effects of iron on growth, biofilm formation, and virulence traits, *Pseudomonas aeruginosa* was cultured in media containing varying concentrations of the iron chelator dipyridyl (DP) (0–400 μM) and FeCl₃ (0–5 μM). Tryptic Soy Broth (TSB) supplemented with 0 μM DP served as the iron-replete environment, with an iron content of 0.65 μg/mL, while TSB with 400 μM DP served as the iron-deficient environment, with an iron content of 0.04 μg/mL. In the environment of TSB supplemented with 400 μM DP and 5 μM FeCl₃, the iron content was 0.37 μg/mL.

### Effect of iron on *Pseudomonas aeruginosa* growth

**Figure 1:**
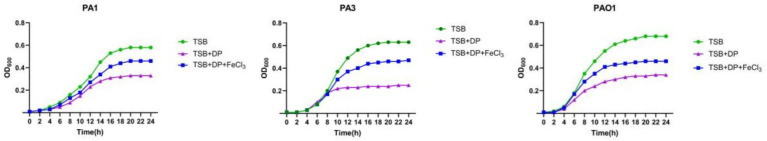
Growth curve of *P. aeruginosa*. All *P. aeruginosa* isolates grew optimally in TSB broth, superior to the growth in TSB broth containing 400 μM iron chelator +(−) 5 μM iron (*p* < 0.05). DP: 2,2′-Dipyridyl, added as an iron chelator. TSB + 400 M DP group was used as control.

All *P. aeruginosa* strains demonstrated optimal growth in iron-replete environments (0 μM DP TSB medium), with significantly better growth compared to both the iron-restricted (400 μM DP) and iron-supplemented (400 μM DP + 5 μM FeCl₃) conditions (*p* < 0.05) (Figure 1).

### Effect of iron on *Pseudomonas aeruginosa* biofilm formation

**Figure 2:**
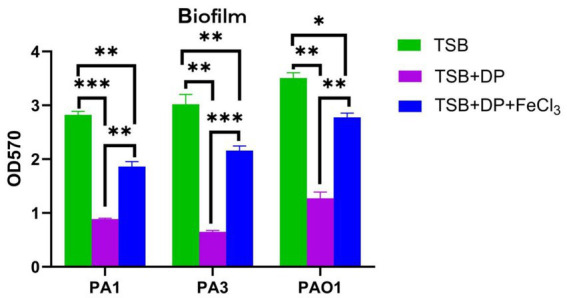
Biofilm formation of *P. aeruginosa.* All *P. aeruginosa* isolates exhibited the strongest biofilm formation ability in TSB broth, which was superior to the biofilm formation ability of the respective strains in TSB broth containing 400 μM iron chelator +(−)5 μM iron (*p* < 0.05). DP: 2,2′-ipyridyl, added as an iron chelator. TSB + 400 M DP group was used as control. Data are presented as mean ± s.d. A student’s t-test was used for between-group comparisons, and multi-group comparisons were performed using analysis of variance (ANOVA). The results are representative of three independent experiments.*p < 0.05; **p < 0.01; ***p < 0.001.

All *P. aeruginosa* strains exhibited significantly enhanced biofilm formation in the iron-replete environment (0 μM DP TSB medium) compared to both the iron-supplemented (400 μM DP + 5 μM FeCl₃) and iron-restricted (400 μM DP TSB) conditions (*p* < 0.05) (Figure 2).

### Effect of iron on virulence determinants of *Pseudomonas aeruginosa*

All *P. aeruginosa* strains exhibited significantly reduced production of virulence determinants—including protease, pyocyanin, exotoxin A, phospholipase C, alkaline protease, elastase, siderophore, and hemolysin—in the iron-replete TSB medium (0 μM DP) compared to both the iron-supplemented (400 μM DP + 5 μM FeCl₃) and iron-restricted (400 μM DP) conditions (*p* < 0.05) (Table 1). Furthermore, the production of these virulence determinants were significantly lower under the iron-supplemented condition (400 μM DP + 5 μM FeCl₃) compared to the iron-restricted condition (400 μM DP).

**Table 1:** Virulence factors of *P. aeruginosa* under iron-restricted and iron-replete culture conditions.

Virulence factors^a^	*P. aeruginosa* isolate PA1			*P. aeruginosa* isolate PA3			*P. aeruginosa* standard strain PAO1		
	TSB	TSB + DP	TSB + DP + FeCl_3_	TSB	TSB + DP	TSB + DP + FeCl_3_	TSB	TSB + DP	TSB + DP + FeCl_3_
Pyocyanin (μg/mL)	0.96 ± 0.01	3.05 ± 0.10	1.64 ± 0.01	0.82 ± 0.01	3.17 ± 0.09	1.65 ± 0.03	1.09 ± 0.03	3.83 ± 0.02	2.22 ± 0.06
Protease (U/L)	1.09 ± 0.01	2.73 ± 0.09	1.65 ± 0.03	1.17 ± 0.03	2.14 ± 0.11	1.55 ± 0.02	1.24 ± 0.06	2.96 ± 0.05	1.83 ± 0.04
Elastase (U/L)	2.02 ± 0.06	5.35 ± 0.08	3.87 ± 0.10	2.56 ± 0.10	7.66 ± 0.21	4.89 ± 0.03	2.97 ± 0.07	7.09 ± 0.36	5.39 ± 0.13
Hemolysin (OD450)	0.25 ± 0.00	0.53 ± 0.01	0.36 ± 0.00	0.16 ± 0.00	0.44 ± 0.01	0.22 ± 0.00	0.23 ± 0.00	0.49 ± 0.01	0.31 ± 0.01
Siderophore (%)	−3.3 ± 0.3	57.9 ± 0.5	47.8 ± 0.4	−4.6 ± 0.4	51.3 ± 0.4	42.0 ± 0.6	−6.8 ± 0.2	64.7 ± 1.3	49.2 ± 0.7
Alkaline Protease (U/gprot)	7.0 ± 0.80	38.0 ± 2.2	16.4 ± 1.5	6.9 ± 1.1	30.9 ± 2.9	18.3 ± 1.2	17.9 ± 0.1	65.9 ± 2.3	31.6 ± 2.1
Phospholipase C (U/gprot)	1.2 ± 0.10	11.3 ± 0.35	2.4 ± 0.10	1.7 ± 0.05	12.0 ± 0.50	2.8 ± 0.15	1.4 ± 0.10	12.6 ± 0.05	3.1 ± 0.35
Exotoxin A (pg/mL)	117.3 ± 11.6	1272.0 ± 26.0	504.7 ± 67.5	959.4 ± 18.4	1824.0 ± 12.0	1101.7 ± 92.5	623.5 ± 54.4	1623.0 ± 28.0	849.2 ± 82.7

^a^The data represents mean ± SD values and is representative of three independent experiments carried out in triplicate.

### *Galleria mellonella* infection model

**Figure 3:**
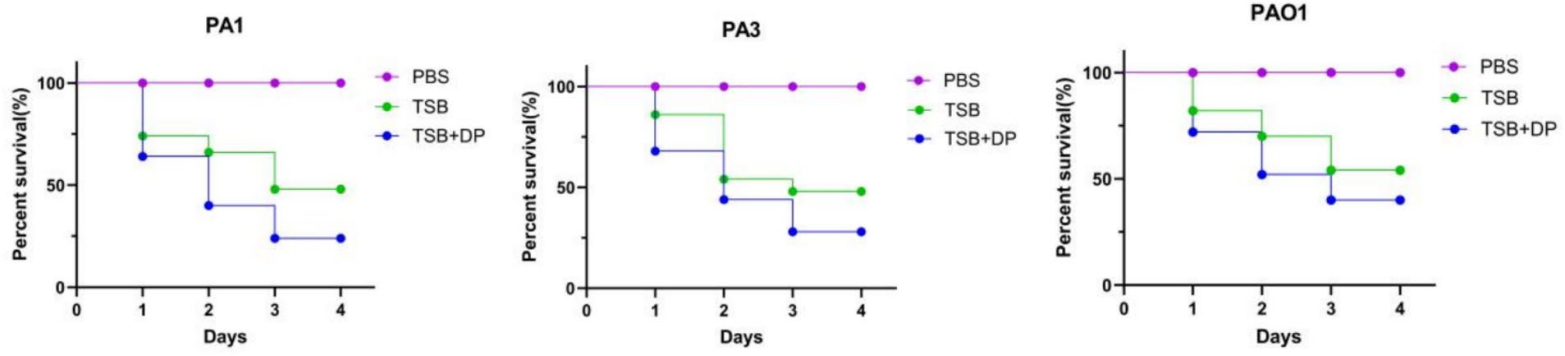
Models of *Galleria mellonella* infected with *P. aeruginosa.* The virulence of PA1, PA3, and PAO1 supplemented with an iron chelator was significantly higher than that of strains without it (*p* < 0.05). DP: 2,2′-Dipyridyl, added as an iron chelator. The PBS was control group. *Galleria mellonella* mortality was analyzed by Kaplan–Meier and log-rank tests.

*Galleria mellonella* larvae infected with *P. aeruginosa* cultured in iron-replete TSB medium (0 μM DP) demonstrated significantly higher survival rates than those infected with bacteria grown in iron-restricted TSB medium (400 μM DP) (Figure 3).

### Pulmonary epithelial cell adhesion assay

**Figure 4:**
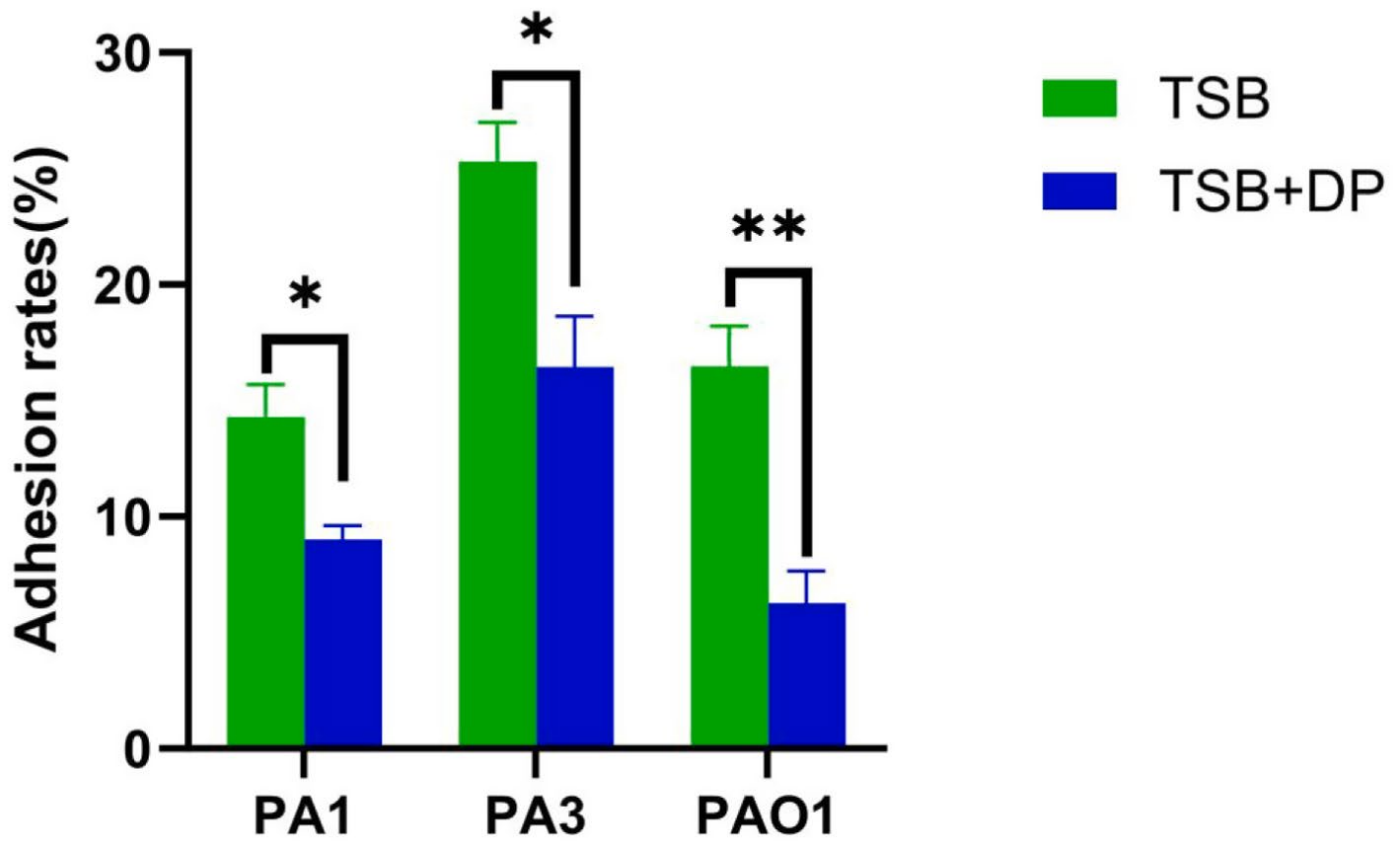
Adhesion capacity of *P. aeruginosa* to pulmonary epithelial cells. All strains exhibited the strongest adhesion capacity in TSB broth, which was superior to the respective strains in TSB broth containing 400 μM iron chelator (*p* < 0.05). DP: 2,2′-Dipyridyl, added as an iron chelator. TSB + 400 M DP group was used as control. A Fisher’s exact test was used for between-group comparisons. Data are presented as mean ± s.d. (*n* = 3).*p < 0.05; **p < 0.01.

*P. aeruginosa* strains cultured under iron-replete conditions exhibited significantly enhanced adhesion to pulmonary epithelial cells compared to those grown in iron-restricted environments (Figure 4).

### Mice pulmonary infection model

#### Bacterial load

**Figure 5:**
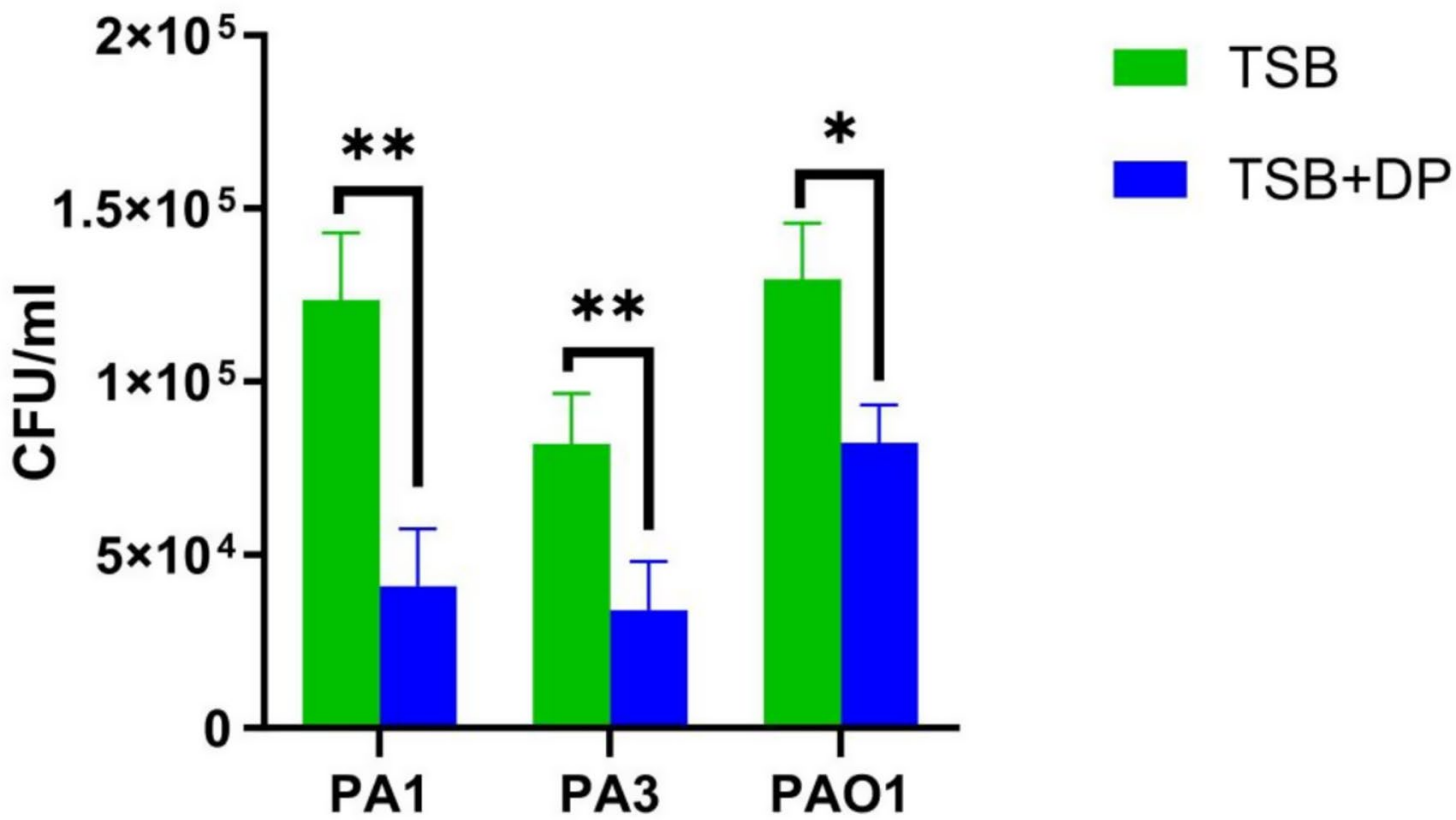
Bacterial load in the lungs of mice infected with *P. aeruginosa.* All strains exhibited a higher bacterial load in mice lungs when cultured in TSB broth than in TSB broth supplemented with 400 μM iron chelator (*p* < 0.05). DP: 2,2′-Dipyridyl, added as an iron chelator. TSB + 400 μM DP group was used as control. A student’s t-test was used for between-group comparisons. Data are presented as mean ± s.d. (*n* = 3).*p<0.05; **p<0.01.

Consistent with the *in vitro* growth observations, mice infected with *P. aeruginosa* cultured in iron-replete TSB medium exhibited significantly higher pulmonary bacterial loads compared to those infected with bacteria grown in iron-restricted TSB medium (400 μM DP) (Figure 5).

#### Inflammatory cytokines

**Figure 6:**
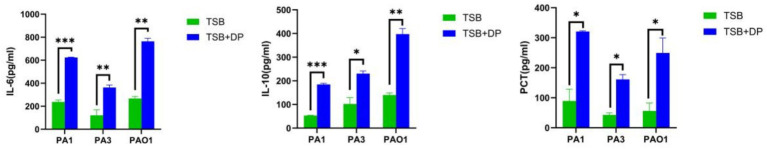
Inflammatory cytokines in the lungs of mice infected with *P. aeruginosa.* All strains exhibited lower levels of inflammatory factors in mice lungs when cultured in TSB broth than in TSB broth supplemented with 400 μM iron chelator (*p* < 0.05). DP: 2,2′-dipyridyl, added as an iron chelator. TSB + 400 μM DP group was used as control. A student’s *t*-test was used for between-group comparisons. Data are presented as mean ± s.d. (*n* = 3). *p<0.05; **p<0.01; ***p<0.001.

Mice infected with *P. aeruginosa* cultured under iron-replete conditions demonstrated significantly lower levels of pulmonary PCT, IL-6, and IL-10 compared to those infected with bacteria grown in iron-restricted medium (Figure 6).

#### Pathological injury

**Figure 7:**
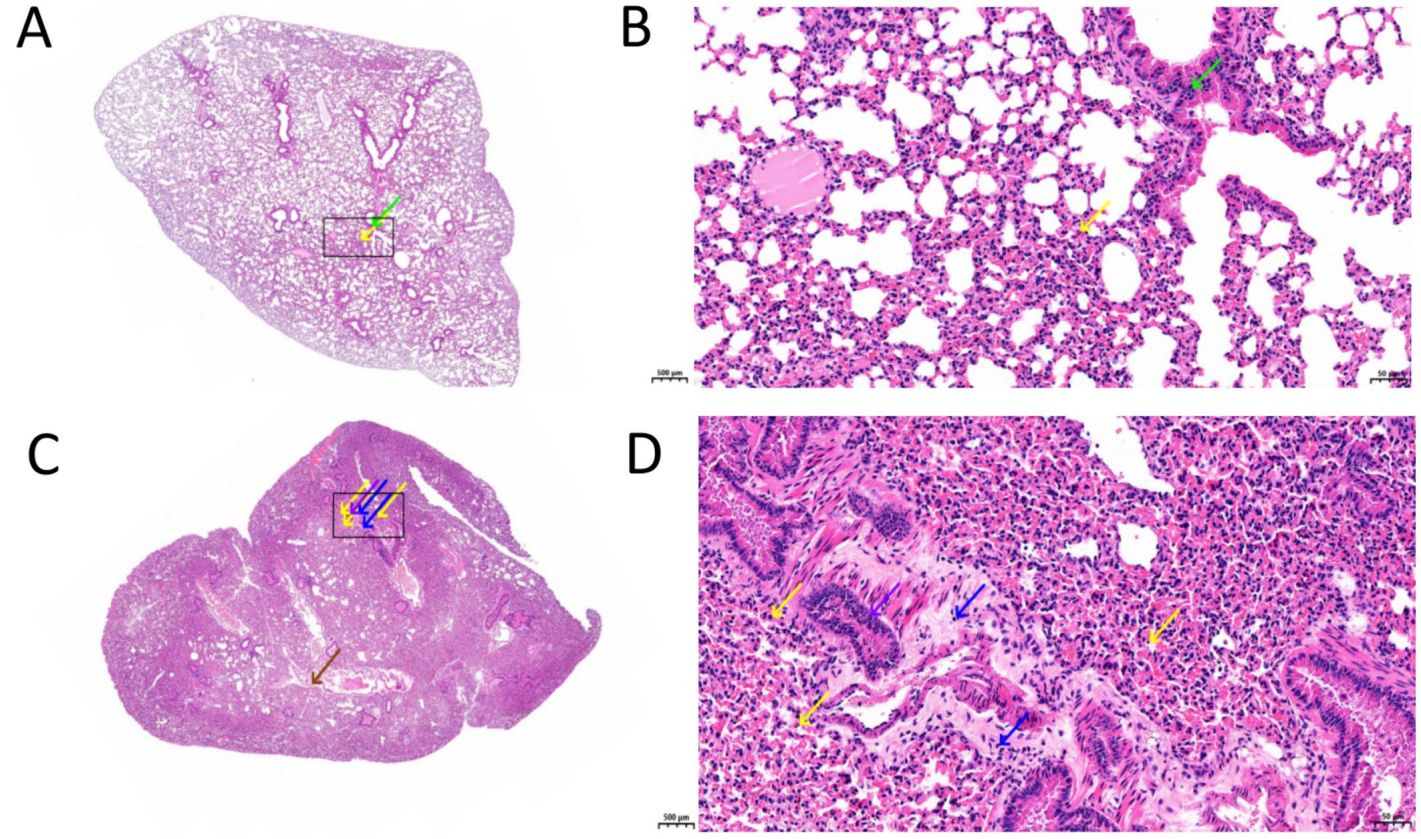
Lung tissues from mice infected with *P. aeruginosa* cultured under different iron conditions were sectioned and subjected to histopathological examination with H&E staining. For mice infected with *P. aeruginosa* cultured in iron-replete TSB medium, overview of lung tissue sections (magnification ×2.0) showed mild overall inflammation **(A)**. The black box indicates the area of the magnified view. At higher magnification (×20.0), a small number of granulocytes were observed infiltrating the alveolar walls (yellow arrows). Minor irregularities were noted in the bronchiolar epithelial cell arrangement, with exfoliated epithelial cells and eosinophilic material visible in the lumen (green arrows) **(B)**. In contrast, lung tissues from mice infected with *P. aeruginosa* cultured in iron-restricted TSB medium (with 400 μM DP) exhibited alveolar narrowing or even obliteration, occasional perivascular hemorrhage (brown arrows), and some interstitial vascular congestion (pink arrows) **(C)**. The black box indicates the area of the magnified view. At higher magnification (×20.0), substantial infiltration of lymphocytes, granulocytes, and macrophages was evident (yellow arrows). Additionally, infiltration by a smaller number of lymphocytes and granulocytes was observed (blue arrows), and eosinophilic material was visible in the bronchiolar lumen (purple arrows) **(D)**. DP: 2,2′-Dipyridyl, added as an iron chelator.

Mice infected with *P. aeruginosa* cultured in iron-replete TSB medium showed minimal granulocyte infiltration in alveolar walls (yellow arrows) and mildly irregular bronchiolar epithelium with luminal exfoliated cells and eosinophilic material (green arrows) (Figure 7B), with black boxes indicating magnified view locations (Figure 7A); whereas those infected with bacteria from iron-restricted medium (400 μM DP) exhibited extensive pathology including alveolar narrowing/collapse (Figure 7C), significant lymphocyte/granulocyte/macrophage infiltration (yellow arrows), bronchiolar irregularities with eosinophilic material (purple arrows), perivascular edema with loose connective tissue (blue arrows), occasional perivascular hemorrhage (brown arrows), and interstitial vascular congestion (pink arrows) (Figure 7D).

## Discussion

The complex pulmonary environment is known to contain varying levels of electrolytes, particularly iron, which has been demonstrated to play a crucial role in determining the ultimate outcome of infections (Chen et al., 2020). Iron exhibits extremely low solubility, and the host restricts its availability to pathogens through complexation with proteins such as transferrin and lactoferrin. This mechanism represents an important host defense strategy (Mayneris-Perxachs et al., 2022). The iron concentration in the pulmonary environment of healthy individuals ranges from 8.6 to 21.5 μg/L, which is relatively close to the iron-restricted condition established in this study. This indicates that the lung is indeed an iron-limited environment under normal physiological conditions, and our study has realistically simulated the pulmonary iron milieu (Gray et al., 2010). Researcher has found that the iron content in the sputum of patients with chronic inflammatory lung diseases (such as cystic fibrosis and bronchiectasis) is significantly elevated compared to that of healthy controls (Gray et al., 2010). Similarly, other study has reported that the iron concentration in the bronchoalveolar lavage fluid of cystic fibrosis patients is also significantly higher than in healthy controls (Ghio et al., 2013). Elevated sputum iron concentration is a characteristic alteration of the pulmonary microenvironment in cystic fibrosis patients. During stable periods, it strongly correlates with the burden of *P. aeruginosa*, suggesting that iron accumulation may promote the persistence of infection (Reid et al., 2007). In the present study, iron-restricted conditions were established by supplementing Tryptic Soy Broth (TSB) with 400 μM dipyridyl, while unsupplemented TSB served as the iron-replete condition. *P. aeruginosa* was cultured separately in iron-restricted and iron-replete media, and its growth, biofilm formation, and pathogenicity under these two conditions were evaluated both *in vitro* and *in vivo*. Previously, study has demonstrated the crucial role of iron in *Klebsiella pneumoniae*-induced liver abscesses by pre-culturing the bacteria under two conditions: iron-replete conditions in TSB and iron-restricted conditions established by adding 300 μM dipyridyl to TSB (Chen et al., 2020).

In this study, we demonstrated that adequate iron availability promoted the growth of *P. aeruginosa*, whereas the isolates exhibited slower growth under iron-restricted conditions. Furthermore, iron enhanced biofilm formation by *P. aeruginosa,* while iron limitation significantly compromised this capacity. Existing studies have demonstrated that iron chelators, including ethylenediaminetetraacetic acid, diethylenetriaminepentaacetic acid, ethylenediamine-*N, N′*-diacetic acid, lactoferrin, deferoxamine, 5-hydroxy-2-hydroxymethyl-4H-pyran-4-one, 3-hydroxy-2-methyl-4-pyrone, 1,2,3,4,6-penta-O-galloyl-*β*-D-glucopyranose, and deferiprone, can significantly inhibit the growth and biofilm formation of *P. aeruginosa* and/or other bacteria and fungi (O’May et al., 2009; Temel and Aksoyalp, 2023; Moon et al., 2013; Nazik et al., 2015; Brilhante et al., 2021; Saha et al., 2025; Guedes et al., 2023; Ramage et al., 2007; Lin et al., 2012; Coraça-Huber et al., 2018; De Sá Almeida et al., 2020; Qiao et al., 2018; Leitão et al., 2025). Furthermore, iron chelators such as deferiprone and deferoxamine can enhance the antibacterial activity of conventional antibiotics like clindamycin, gentamicin, vancomycin, amoxicillin-clavulanic acid, and meropenem by disrupting bacterial biofilms (Temel and Aksoyalp, 2023). The reduced biofilm formation by *P. aeruginosa* under iron-restricted conditions may be associated with the decreased biosynthesis of *Psl* exopolysaccharide, a key component of its biofilm, which is elevated under iron-replete conditions (Yu et al., 2016). Previous studies have confirmed that *Acinetobacter baumannii* similarly shows markedly diminished biofilm formation in iron-limited environments, with significant restoration upon iron supplementation, indicating iron’s essential role in *A. baumannii* biofilm development (Saha et al., 2025). The presence of sufficient iron also facilitates biofilm formation in *Staphylococcus epidermidis, Staphylococcus aureus,* and *Klebsiella pneumoniae* (Chen et al., 2020; Oliveira et al., 2017; Lin et al., 2012). Collectively, these findings and our results substantiate that iron significantly modulates bacterial growth and biofilm formation processes.

Previous studies have established that iron promotes *Klebsiella pneumoniae* virulence through enhanced serum resistance, potentially mediated by iron uptake genes *sitA/C/D*, prompting our investigation into iron’s role in modulating *P. aeruginosa* virulence during pulmonary infection (Chen et al., 2020; Chen et al., 2025). Our systematic assessment under defined iron conditions revealed maximal production of all virulence determinants (proteases, exotoxin A, phospholipase C, alkaline protease, elastase, siderophores, and hemolysins) under iron restriction, with minimal yields in iron-replete TSB, while observing enhanced BEAS-2B pulmonary epithelial adhesion in iron-rich conditions. These findings align with Kim and Michael, who similarly reported upregulated protease, exotoxin A, and pyochelin production in *P. aeruginosa* under iron-depleted conditions (TSB + DP) (Kim et al., 2003; Bjorn et al., 1979). Additionally, we found that under iron-deficient conditions, hemolysin production in *P. aeruginosa* was significantly increased. Mechanistically, iron deficiency induces adaptive stress responses in strain PAO1, including significantly elevated secretion of elastase, hemolysin, and siderophores, which facilitates bacterial iron acquisition (Schaible et al., 2017). Furthermore, *Fur*, the master regulator of iron homeostasis in *P. aeruginosa*, controls the expression of hundreds of genes, including those involved in iron acquisition and virulence. The *Fur* protein represses the expression of genes responsible for the biosynthesis and transport of the siderophores pyoverdine and pyochelin. As a result, these iron-chelating compounds are produced exclusively under iron-limited conditions (Sánchez-Jiménez et al., 2023). Similarly, under iron-sufficient conditions, *Fur* represses the expression of the hemophore *HasAp* and the heme uptake systems *Phu*, *Has*, and *Hxu*. Furthermore, *Fur*-mediated repression encompasses other key virulence factors, such as exotoxin A, the proteases *PIV/PrpL* and *IcmP*, the extracellular lipase *LipA*, and the hemolytic phospholipase C *PlcH* (Ochsner et al., 2002). *Galleria mellonella* larvae, an invertebrate model, are commonly used for assessing bacterial virulence (Wang et al., 2022; Insua et al., 2013). Complementary *Galleria mellonella* infection models confirmed significantly higher larval mortality with iron-restricted (TSB + DP) versus iron-replete (TSB) cultures, collectively substantiating that iron availability critically governs *P. aeruginosa* virulence expression.

To further evaluate *in vivo* the characteristics of enhanced adhesion and attenuated virulence of *P. aeruginosa* under iron-replete conditions, we employed a murine pulmonary infection model, which shares structural and genetic similarities with human lungs (Mural et al., 2002). Mice were infected with *P. aeruginosa* cultured in either iron-restricted or iron-replete media. Infection with bacteria grown under iron-replete conditions resulted in significantly higher pulmonary bacterial loads, alongside markedly reduced pathological damage and inflammatory cytokine levels. Conversely, mice infected with *P. aeruginosa* from iron-restricted medium exhibited significantly lower bacterial counts but intensified pathological injury and inflammatory responses. These *in vivo* results may be attributed to the fact that, under *in vitro* iron-restricted conditions, *P. aeruginosa* exhibits reduced adhesion to pulmonary epithelial cells while concurrently increasing the production of siderophores, pyocyanin, alkaline protease, exotoxin A, elastase, protease, phospholipase C, and hemolysins. Existing study has demonstrated that the siderophores pyoverdine and pyochelin of *P. aeruginosa* play distinct roles in the virulence of acute pulmonary infections. Pyoverdine induces cell death by modulating rhamnolipid secretion, independently of host iron chelation or known toxins. Pyochelin can penetrate cell membranes, activating pro-inflammatory pathways (such as NLRP3 inflammasome and IL-8 secretion) by chelating intracellular iron, with pyoverdine enhancing this pro-inflammatory effect (Donghoon et al., 2024). Furthermore, in a mouse pneumonia model, it has been confirmed that siderophores (enterobactin, salmochelin, and yersiniabactin) secreted by *Klebsiella pneumoniae* can independently induce the secretion of inflammatory cytokines including IL-6, CXCL1, and CXCL2, promote bacterial dissemination to the spleen, and stabilize host hypoxia-inducible factor-1α (HIF-1α), which in alveolar epithelial cells is a prerequisite for bacterial dissemination (Holden et al., 2016).

Previous studies have demonstrated that iron availability in the *in vitro* culture environment can alter the renal pathogenicity of *Escherichia coli* in mice (S et al., 1991). However, there remains a paucity of correlated research examining *P. aeruginosa* under different iron conditions in the context of pulmonary infection. To address this gap, we pre-cultured *P. aeruginosa* in iron-restricted and iron-replete media prior to inducing murine pulmonary infection, thereby elucidating its iron-dependent pathogenicity *in vivo*. Our findings indicate that iron levels influence the growth, biofilm formation, and virulence traits of *P. aeruginosa*, establishing its crucial role in pathogenicity.

Currently, the siderophore-based antibacterial agent cefiderocol, which targets bacterial iron metabolism, has demonstrated remarkable efficacy against resistant Gram-negative bacteria such as *P. aeruginosa* (Cruz-López et al., 2022). Additionally, the FDA-approved iron chelators deferoxamine and deferasirox, by restricting *P. aeruginosa*’s ability to acquire iron from the environment, have been shown to reduce existing *P. aeruginosa* biofilm production by approximately 90% and significantly decrease viable bacterial counts when used in combination with tobramycin (Moreau-Marquis et al., 2009). Based on the findings of this study, iron is demonstrated to be essential for the growth, biofilm formation, and pathogenicity of *P. aeruginosa* in the context of pulmonary infection. However, attempting to treat these infections by creating an iron-restricted condition solely through the use of iron chelators such as deferoxamine and deferasirox may potentially exacerbate lung damage in patients. This is because our study also revealed that under iron-deficient conditions, key virulence determinants of *P. aeruginosa*—including siderophores, pyocyanin, proteases, hemolysins, phospholipase C, and exotoxin A—were significantly elevated (Table 1). Additionally, inflammatory markers such as PCT, IL-6, and IL-10 showed significant increases (Figure 6). Most critically, lung injury in mice was more pronounced under these conditions (Figure 7).

Pyoverdine, a major siderophore and a key virulence factor in *P. aeruginosa*, plays a critical functional role. It not only facilitates iron acquisition but also regulates the secretion of exotoxin A, endoprotease, and pyoverdine itself (Lamont et al., 2002). Bacterial efflux pumps, such as those from the RND family, typically mediate cellular adaptive responses to various environmental stimuli, including iron. Hannauer et al. discovered that one of the pathways by which *P. aeruginosa* secretes the siderophore pyoverdine is via efflux pumps (Hannauer et al., 2010). Efflux pump inhibitors, by interfering with iron uptake and simultaneously reducing the secretion of key virulence factors like pyoverdine, may represent a promising therapeutic strategy for managing persistent pulmonary infections (Mudgil et al., 2024). Gallium nitrate, an iron metabolism disruptor, has been shown to inhibit the growth and biofilm formation of *P. aeruginosa*. However, pyoverdine can sequester gallium nitrate within the periplasmic space through irreversible chelation, significantly diminishing its antibacterial activity. 5-fluorocytosine can overcome this antagonism by blocking either the synthesis or function of pyoverdine, thereby enabling synergistic efficacy with gallium nitrate for potent antibacterial and virulence-attenuating effects, while maintaining a low selective pressure for resistance development (Kang et al., 2021). Additionally, alternative anti-virulence strategies employing natural compounds, such as Manuka honey and allicin, target siderophore systems. By suppressing pyoverdine production, they disrupt bacterial iron acquisition, concurrently inhibiting biofilm formation and the expression of virulence factors (Kronda et al., 2013; Xu et al., 2019). Owing to their lower cytotoxicity to host cells, these strategies hold promise for the clinical treatment of *P. aeruginosa* infections.

## Limitations

This study has several limitations. First, it was conducted at a single center, which may limit the diversity and representativeness of the bacterial strains included. Additionally, all experiments assessing growth, biofilm formation, and virulence were performed exclusively on *P. aeruginosa* in the planktonic mode; these results may not fully reflect the responses of *P. aeruginosa* in the biofilm mode. The specific pathways through which iron affects the growth, biofilm formation, and pathogenicity of *P. aeruginosa* and contributes to persistent pulmonary infections remain to be further investigated. Furthermore, in this study, the use of the iron chelator to create iron-restricted conditions in tryptic soy broth (TSB), and the use of TSB alone as the iron-replete condition, may not fully recapitulate the complexity of the lung environment. Despite these limitations, our findings establish a foundation for a deeper understanding of the role of iron in the pathogenesis of *P. aeruginosa* pulmonary infection.

## Conclusion

In summary, environmental iron facilitates the growth and biofilm formation of *P. aeruginosa* causing pulmonary infections, while attenuating its virulence. This iron-mediated adaptation may be associated with the persistence of *P. aeruginosa* pulmonary infections. To better understand these findings, a further in-depth evaluation is warranted.

## Data Availability

The original contributions presented in the study are included in the article/Supplementary material, further inquiries can be directed to the corresponding authors.
