# Supplementary figures and images for: Iron dictates the growth, biofilm formation, and virulence of Pseudomonas aeruginosa in pulmonary infections
Source: Front Microbiol. 2026 Jan 16;16:1742683. doi: 10.3389/fmicb.2025.1742683 (PMC12855490; doi:10.3389/fmicb.2025.1742683)

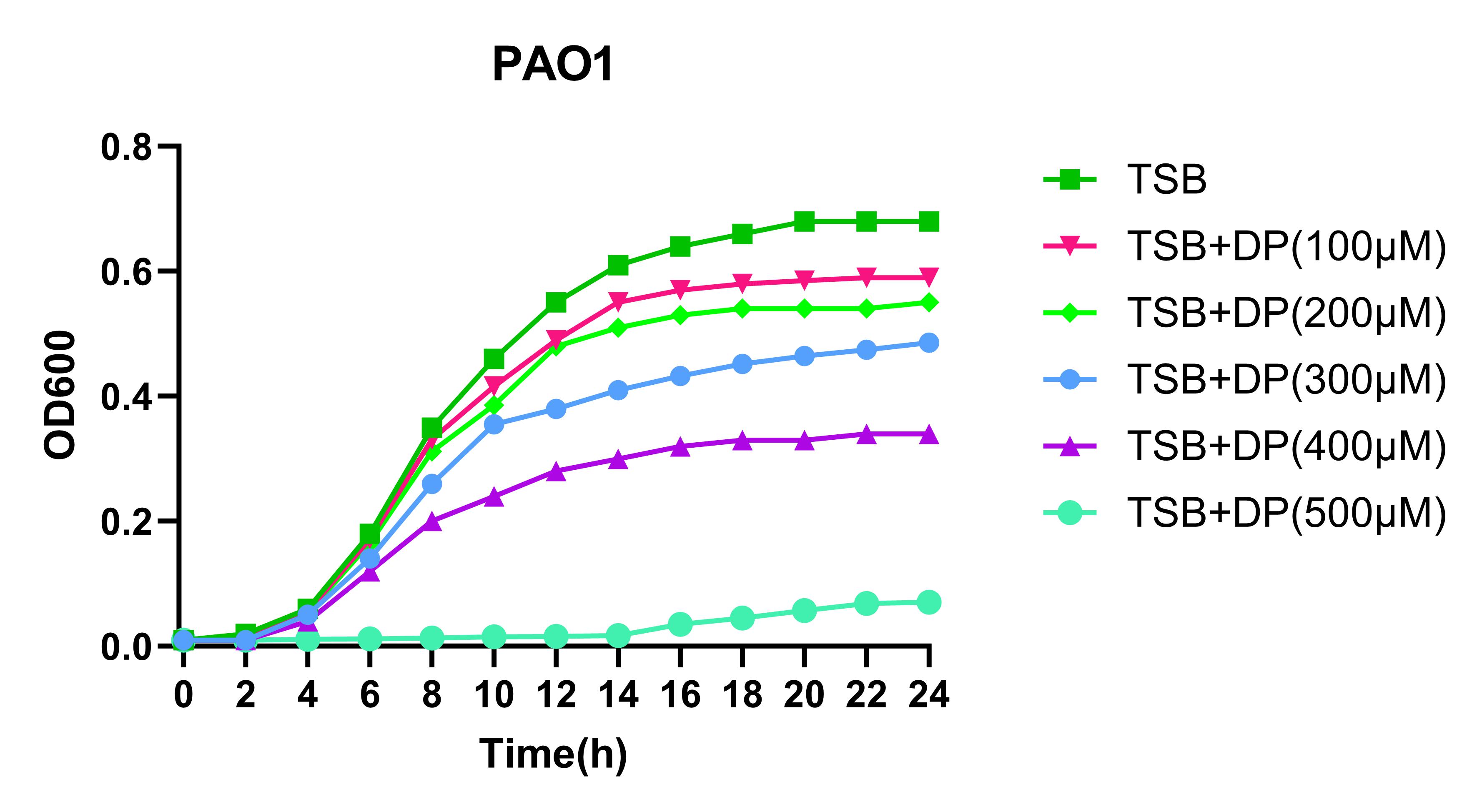

Supplement: Supplementary file 1 [file Image_1.jpeg]

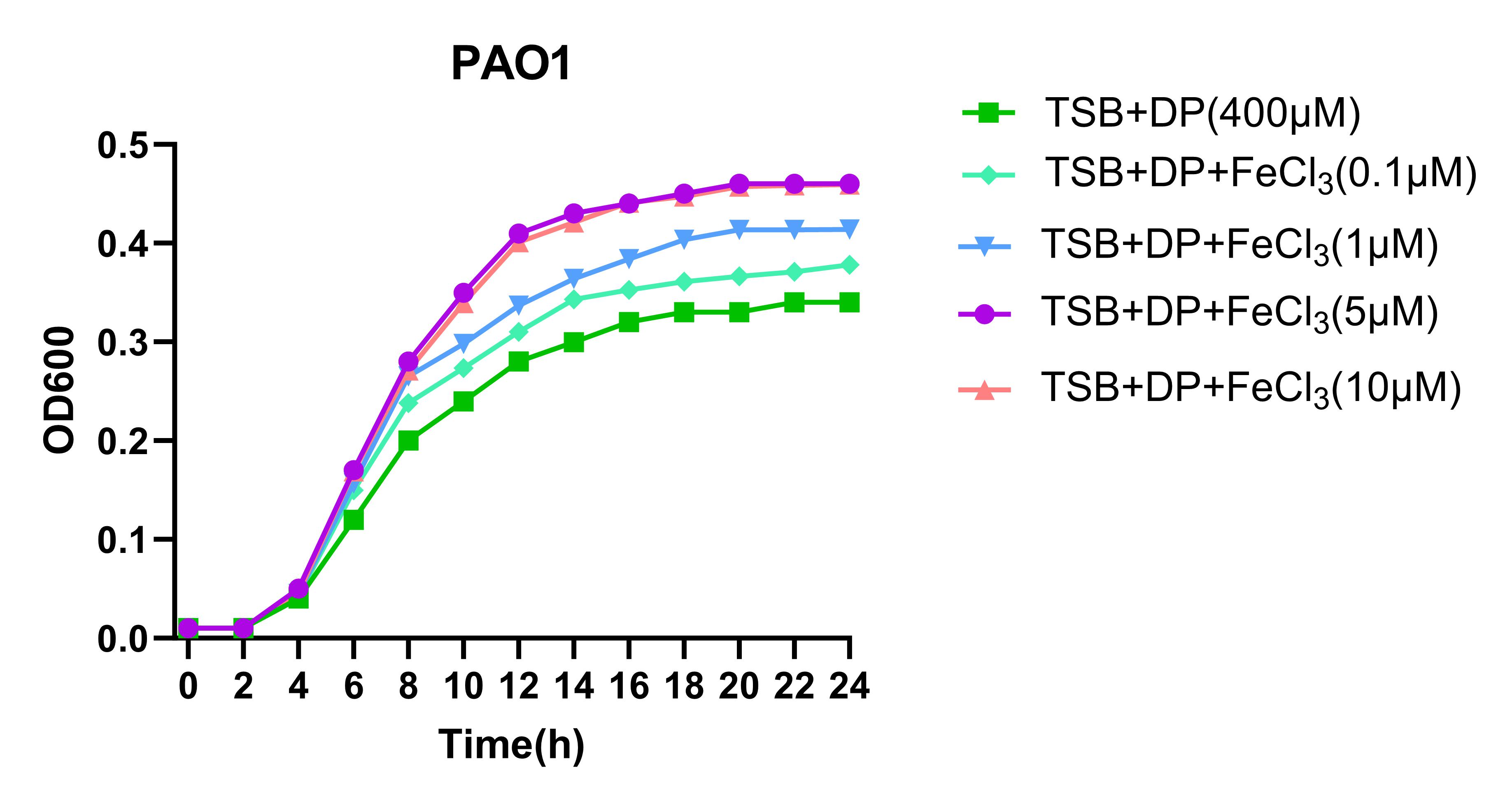

Supplement: Supplementary file 2 [file Image_2.jpeg]
